# Low Ppm Atom Transfer Radical Polymerization in (Mini)Emulsion Systems

**DOI:** 10.3390/ma13071717

**Published:** 2020-04-06

**Authors:** Karolina Surmacz, Paweł Chmielarz

**Affiliations:** 1 Doctoral School of Engineering and Technical Sciences at the Rzeszow University of Technology, Al. Powstańców Warszawy 8, 35-959 Rzeszów, Poland; d503@stud.prz.edu.pl; 2 Department of Physical Chemistry, Faculty of Chemistry, Rzeszow University of Technology, Al. Powstańców Warszawy 6, 35-959 Rzeszów, Poland

**Keywords:** low ppm ATRP, dispersed media, miniemulsion, well-defined polymers

## Abstract

In the last decade, unceasing interest in atom transfer radical polymerization (ATRP) has been noted, especially in aqueous dispersion systems. Emulsion or miniemulsion is a preferred environment for industrial polymerization due to easier heat dissipation and lower production costs associated with the use of water as a dispersant. The main purpose of this review is to summarize ATRP methods used in emulsion media with different variants of initiating systems. A comparison of a dual over single catalytic approache by interfacial and ion pair catalysis is presented. In addition, future development directions for these methods are suggested for better use in biomedical and electronics industries.

## 1. Introduction

**Scheme 1:**
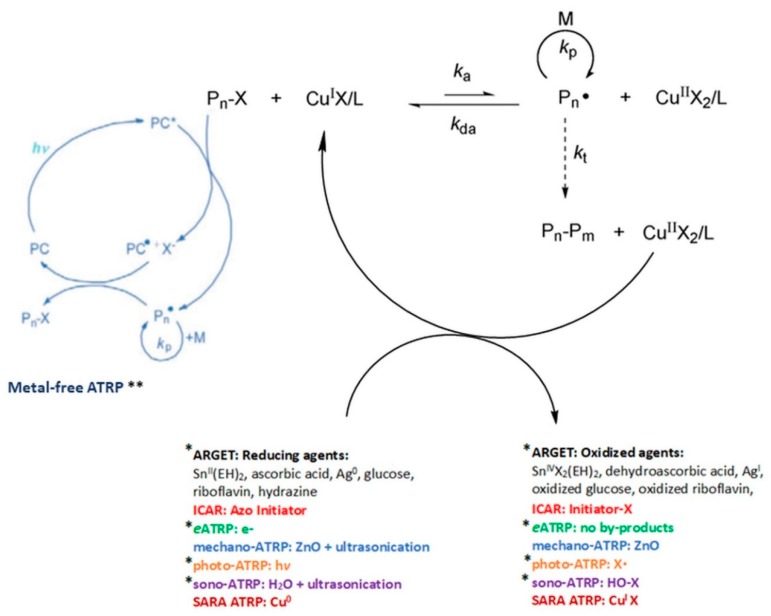
ATRP mechanisms with low ppm catalysts concentrations (* methods described in review, ** proposed future direction).

Nowadays, atom transfer radical polymerization (ATRP) is one of the most certain and versatile route for preparing well-defined polymers with precise topology and architecture [1,2,3,4,5]. Initially, copper(I) or other transition-metals (e.g., ruthenium [6], iron [7]) were employed in the catalyst system as mediators of reversible activation/deactivation between the dormant species and propagating radicals, assuring control over the polymerization process [8,9,10,11]. One of the main disadvantages of these methods was the necessity for high catalyst concentrations loading, which was improved by the addition of an additional redox cycle, thus decreasing the catalyst concentration to a “low ppm” level [12,13,14,15]. The development of “low ppm” ATRP techniques [16] comprise an activator regeneration by electron transfer (ARGET) ATRP [17,18,19,20], initiator for continuous activator regeneration (ICAR) ATRP [21,22,23,24], supplemental activators and reducing agents (SARA) ATRP [25,26,27,28,29], and external stimuli induced approaches—mechanically induced ATRP (mechano-ATRP) [30,31], photoinitiated ATRP (photo-ATRP) [32,33,34,35], electrochemically mediated ATRP (*e*ATRP) [36,37,38,39,40,41,42], ultrasonication-induced ATRP (sono-ATRP) [5,43] and metal-free ATRP [44,45,46] (Scheme 1).

ATRP techniques in dispersed media are especially beneficial for obtaining multifunctional polymers with narrow molecular weight distribution (MWD, *M*_w_/*M*_n_, *Đ*) and a predetermined molecular weight (MW), as segregation and compartmentalization decrease the rate of termination processes [47,48,49]. Polymerizations could be carried out in various media, including homogeneous systems (bulk or solution) or biphasic heterogeneous systems (suspension, emulsion, miniemulsion) [50]. The biphasic heterogeneous systems contain a mixture of two immiscible liquids [51]. The industrial polymerizations are mainly carried out in a normal oil-in-water (O/W) system or an inverse water-in-oil (W/O) system [52,53]. Various polymer structures, including block [27,54], star [55,56,57] and brush [58,59,60], were synthesized in water-dispersed media (microemulsion [61,62,63], miniemulsion [64,65,66], and emulsion [67,68,69,70]). Emulsion and miniemulsion methods belong to a heterogeneous environment with water as a main dispersing medium for polymerization of insoluble or low-soluble monomers to receive dispersed nanoparticles [71]. Water, as a continuous phase, enables excellent heat transfer, ensures lower viscosity during polymerization, and makes these techniques eco-friendly [71]. Furthermore, droplets in emulsion act as tiny reactors that limit the amount of reactive monomer necessary to form the particle. The use of water ensures the synthesis of hydrophobic polymers at a low cost [38,72,73]. 

Miniemulsion and emulsion polymerizations generally produce polymer latexes in a size range of 50–500 nm [34]. Considering miniemulsion nanoparticles, surfactant and ultrasonic homogenization are used to break down the monomer droplets to reach a metastable heterogeneous state and reduce Ostwald ripening (transfer of monomer from small to large droplets aims to reduce total surface energy of the system) [74]. Although the miniemulsion has many similarities to emulsion polymerization, their distinct features concern particle nucleation and mass transportation. Miniemulsion micelles are not present in micron sizes [75], because after sonication the system contains only droplets of submicron monomer with relative stability [74]. Before sonication, oil-soluble initiators are mixed with monomers so that the submicron droplets encapsulate all necessary reactants (without dilution effect) followed by initiation and polymerization without any mass transfer through a continuous media. This feature provides unique advantages of miniemulsion, such as constant (or insignificantly changed) viscosity during synthesis and easier removal of the resulting product. However, generating stable miniemulsion droplets requires high energy input during the sonication process, limiting its broad application in many scale-up productions in the industry [74]. The miniemulsion polymerization is a versatile technique for the formation of a broad range of structured materials as linear or branched polymers with predetermined molecular weight and low dispersity [34,38,43].

## 2. Electrochemically Mediated Atom Transfer Radical Polymerization (*e*ATRP)

**Scheme 2:**
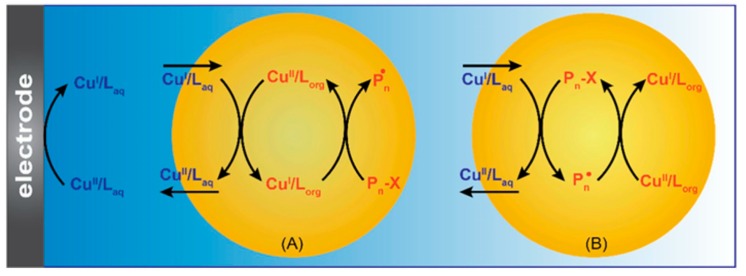
Mechanism of a dual catalysis in miniemulsion *e*ATRP. Pathway (A)—reduction of Cu^II^/L_org_, then chain end activation by Cu^I^/L_org_; pathway (B)—direct activation of the chain end. Reprinted with permission from reference [76], 2016, American Chemical Society.

An electrochemically mediated approach was successfully applied to mediate ATRP in dispersed media (miniemulsion) of *n*-butyl acrylate (*n*BA) [38,76]. In *e*ATRP, reduction of X−Cu^II^/L to Cu^I^/L requires a cathodic current flowing from the metal working electrode (WE) and occurs at the electrode surface [37,77,78]. The rate and control over the polymerization are adjusted by the electrochemical parameters of the system, such as current intensity (*I*_app_) or applied potential (*E*_app_) [41,79,80]. The first concept involved the use of a dual catalytic system composed of two distinct copper catalysts, with a hydrophobic and hydrophilic characteristics, soluble in an organic and aqueous phase, respectively [1,50,76,81]. This system (Cu^II^/L_aq_ + Cu^II^/L_org_) provided well-defined poly(*n*-butyl acrylate) by the *e*ATRP approach. Electropolymerization under heterogeneous conditions is difficult because in (mini)emulsion the electrode and reagents are separated. The electrode is in contact with the continuous aqueous phase, whereas polymerization components (monomer, initiator and radicals) are dispersed in the organic phase [76]. Therefore, to initiate the polymerization an electrochemical stimulus must penetrate the aqueous phase and subsequently proceeds to the dispersed phase. Furthermore, miniemulsion *e*ATRP comprises a challenge compared to other organic reactions, because radicals must be continuously activated/deactivated after the electrochemical stimulus reaches the organic phase (Scheme 2).

In this approach, Cu^II^/L_aq_ is reduced at the electrode–water interface. Then, the electrochemical stimulus is passed from the electrode through water to the monomer droplets mainly by Cu^I^/L_aq_ migration. The catalyst partition and interfacial dynamics constitute new significant parameters to regulate the process in miniemulsion *e*ATRP [76].

As opposed to the initial concept, a single hydrophilic complex can also catalyze miniemulsion *e*ATRP, if the cationic copper complex has strong interaction with an anionic surfactant. The catalyst/surfactant system produces ion pairs at the droplet surface to transport the catalyst into monomer droplets, thus enabling a controlled polymerization, enhanced by interfacial catalysis [82,83]. Different combinations of surfactant and hydrophilic Cu complexes were studied in the miniemulsion *e*ATRP of *n*-butyl acrylate [38]. Table 1 shows the results of *e*ATRP of *n*BA in miniemulsion with various surfactants and catalysts.

**Scheme 3:**
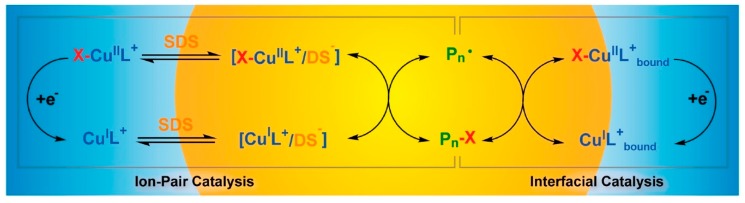
Mechanism of ion-pair and interfacial catalysis in miniemulsion *e*ATRP. Reprinted with permission from reference [38], 2017, American Chemical Society.

The application of an anionic surfactant revealed a specific interaction between X−Cu^II^L and sodium dodecyl sulfate (SDS), and favored polymerization in dispersed monomer droplets, without the necessity of using a dual catalyst (hydrophilic and hydrophobic) combination [65]. The strongly hydrophilic catalyst complex (copper(II) bromide/tris(2-pyridylmethyl)amide Cu^II^Br_2_/TPMA) effectively controlled a miniemulsion *e*ATRP inside stabilized hydrophobic *n*BA droplets. In such a system, the ideal Cu catalyst should have (i) high activity, (ii) the ability to form ionic pairs with the anionic surfactant, and (iii) greater affinity for creating ion pairs in the oxidation state (Cu^II^). Scheme 3 presents the proposed mechanism of *e*ATRP in miniemulsion with the X-Cu^II^TPMA/SDS catalytic system [76]. According to the interfacial catalysis, 95% of catalyst is placed on the interface of the hydrophobic monomer and water, only ≈1% is inside micelles in the form of ion-pairs, while the remaining 4% is in the aqueous phase. The transfer of atoms was carried out *via* interfacial catalysis (catalyst bound to the surface of droplets) as well as by ion-pair catalysis (ion pairs distributed in monomer droplets) [38].

The presence of non-ionic polyoxyethylene(20) oleyl ether (Brij-98) gave an uncontrolled polymerization with broad molecular weight distribution of final products, indicating an insufficient amount of deactivator in the hydrophobic droplets. Actually, *e*ATRP with a single catalyst system (X−Cu^II^TPMA/SDS) was much faster and was slightly better defined in terms of MW and MWD than *e*ATRP with dual catalysis [38,76].

## 3. Activators Regenerated by Electron Transfer Atom Transfer Radical Polymerization (ARGET ATRP)

Initially, it was thought that the key to success in ARGET ATRP in aqueous dispersed systems is strongly dependent on the ligand, which must be both hydrophobic and highly active [84]. Hydrophobicity prevents the catalyst from diffusing to the aqueous phase [84], while high activity, or *K*_ATRP_ value, affords well-controlled polymerizations at low catalyst concentration [85]. The breakthrough in the field of ARGET ATRP in aqueous dispersed media was the design and synthesis of bis[2-(4-methoxy-3,5-dimethyl)pyridylmethyl]octadecylamine (BPMODA*) as a highly active ligand. The modification of previously used bis(2-pyridylmethyl)octadecylamine (BPMODA) [85] was achieved by the addition of electron-donating groups, which resulted in a ca. 100-fold increase in the *K*_ATRP_ value. Heterogeneous polymerizations carried out in the range of catalyst concentrations (2,000–250 ppm) with BPMODA* consistently resulted in increased control over synthesis, particularly during the initial stages of the process (low monomer conversion) [86]. The same research group investigated an application of a tetradentate hydrophobic ligand which was both hydrophobic (*N′,N*″-dioctadecyl-*N′,N*″-bis[2 (4-methoxy-3,5-dimethyl)pyridylmethyl] ethane-1,2-diamine (DOD-BPED*) also of a highly active nature. The Cu^II^Br_2_/DOD-BPED* catalytic system proved to be extremely useful in miniemulsion ARGET ATRP, maintaining control over the polymerization of *n*-butyl methacrylate (*n*BMA) at concentrations of catalyst as low as 50 ppm [87].

**Scheme 4:**
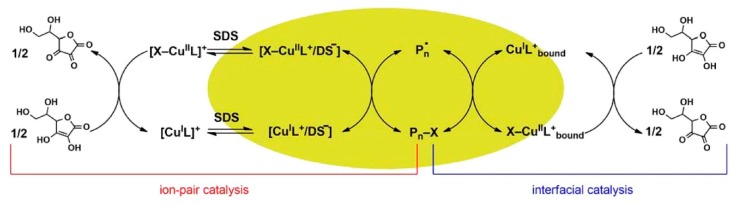
Mechanism of ion-pair and interfacial catalysis in miniemulsion ARGET ATRP with ascorbic acid as a reducing agent. Reprinted with permission from reference [19], 2017, American Chemical Society.

Inspired by the development of the novel catalytic system for *e*ATRP and based on a strongly hydrophilic complex, X−Cu^II^TPMA^+^ [76], the concept of ion-pair and interfacial catalysis in miniemulsion ARGET ATRP of different acrylate monomers, with ascorbic acid (AsAc) as the reducing agent, was exploited. Despite the insolubility of the catalytic complex in *n*BA, it enters hydrophobic droplets in combination with an anionic surfactant [16]. The interaction between the catalytic system and SDS formed inert ion pairs (X−Cu^II^TPMA/SDS) which resided on the surface or inside monomer droplets. In this approach, the final product contained a negligible residue of copper, excluding the need for an additional purification. The hydrophilic complex (X−Cu^II^TPMA^+^) migrated into the water phase while the miniemulsion was breaking up by dilution. The interfacial and ion-pair mechanism of polymerization, identical to miniemulsion *e*ATRP, provided well-defined star and brush polymers, monomodal block copolymers (P*n*BMA-*b*-P*n*BA and P*n*BMA-*b*-P*t*BMA), and P*n*BMA with low dispersity (using only 50 ppm catalyst) [38] (Scheme 4).

Several anionic surfactants were evaluated to form ion-pair complexes: SDS, sodium dodecylbenzenesulfonate (SDBS), and sodium dodecanoate (SDA) [19]. This concept could be considered a paradigm shift for miniemulsion ATRP, which previously required very hydrophobic catalysts that were predominately confined to the organic phase [76,82,86,88].

**Scheme 5:**
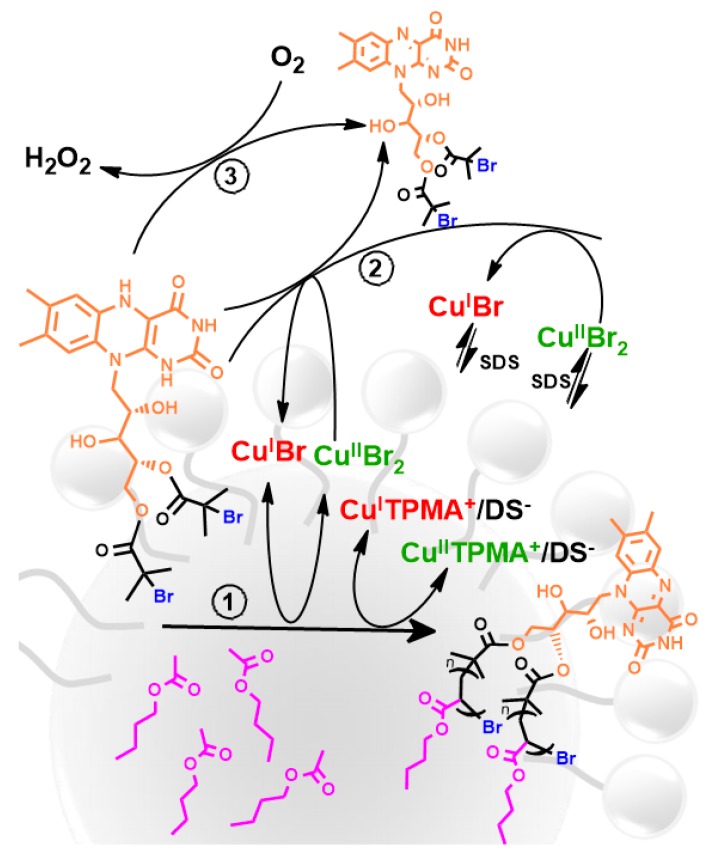
Mechanism of ARGET ATRP in miniemulsion with triple-functional riboflavin-based macroinitiator acting as an initiator (route 1), reducing agent (route 2) and oxygen scavenger (route 3). Reprinted with permission from reference [66], WILEY-VCH Verlag GmbH and Co. KGaA, Weinheim. Copyright 2020.

Currently, the polymer industry is mainly focused on the development of green and sustainable technologies in various polymerization processes. To meet such requirements, our group described the concept of the naturally-derived triple-functional riboflavin-based macromolecule for an efficient ARGET ATRP of acrylate monomer in miniemulsion media in an air tolerant environment [66]. This results in the synthesis of polymer brushes with riboflavin core and PBA side chains characterized by narrow molecular weight distribution. All of the mentioned syntheses by ARGET ATRP approach are summarized in Table 2. The catalysis in miniemulsion ARGET ATRP is described by interfacial and ion-pair intermolecular and intramolecular mode interactions. The riboflavin-based molecule (Rib-Br_2_) with two modified hydroxyl groups in a ribitol tail is located on the surface of micelles (on the phase interface), mimicking a Pickering mechanism and enabling effective polymerization even with only 186 ppm (by weight) of copper catalyst. The functionalized riboflavin supramolecular structure plays three significant functions in preparation of polymers by miniemulsion ARGET ATRP methodology, acting as an initiator due to modified brominated tail of ribitol; a reducing agent due to the preservation of redox functionality of isoalloxazine ring; and an oxygen scavenger, enabling the reaction in air conditions (Scheme 5) [66]. 

## 4. Photoinduced Atom Transfer Radical Polymerization (photo-ATRP)

The implementation of photoinduced processes in aqueous dispersed can provide more ecological approaches to the preparation of polymers. This type of photopolymerization was successfully performed in miniemulsion [34] and emulsion [35] environments. The external regulation of polymerization eliminates additional chemical compounds to activate the reaction and provides an efficient temporal control [89,90,91]. Considering its high energy efficiency and possibilities for spatiotemporal control, the photoinduced ATRP is a suitable candidate for use in industrial production [92].

**Scheme 6:**
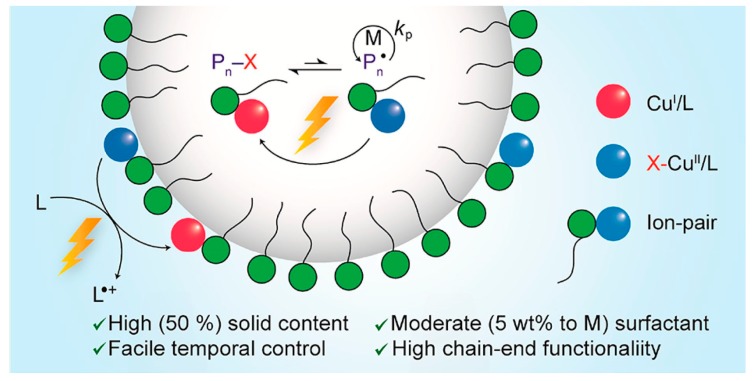
Mechanism of photo-ATRP in miniemulsion. Reprinted with permission from reference [34], 2018, American Chemical Society.

In miniemulsion and emulsionpolymerization, particle sizes are in the range of 50–500 nm [34], which is comparable to the light wavelength. The turbidity and opacity hampers the light penetration; therefore, the effects of light scattering and absorption in the polymerization should be considered (Scheme 6) [34]. The latest advances in photo-ATRP in miniemulsion and emulsion media achieved with UV or LED light are summarized in Table 3.

For a wavelength of 370 nm as a driving force in ATRP in miniemulsion media, a full range of different key parameters in the reaction including a vial size, Cu^II^Br_2_/TPMA ratio, amount of SDS, solids content, and concentration of the Cu catalyst were examined [34].

**Figure 1:**
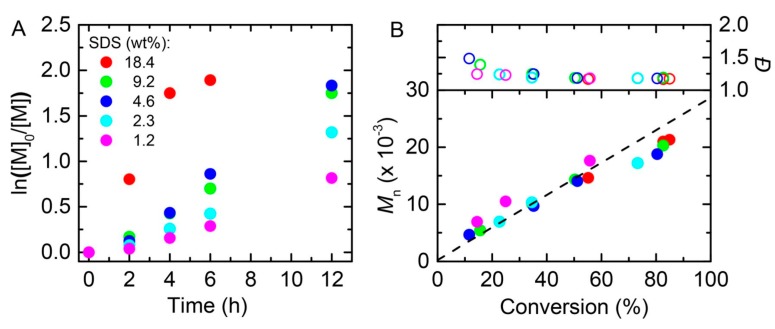
Miniemulsion photo-ATRP of BMA in the presence of various SDS concentrations. (**A**) Kinetic plots; (**B**) Evolution of *M*_n_ and *Đ* over monomer conversion. Reprinted with permission from reference [34], 2018, American Chemical Society.

The ability of light to penetrate through the reaction medium is a factor that limits the speed of photochemical processes. Therefore, the most effective reaction takes place on the surface of the reaction vial where the light absorption is the highest. Another factor directly affecting the light absorption is the particle size. This parameter could be adjusted by varying the amount of SDS surfactant. Coagulation after polymerization was observed when the amount of SDS was 1.2 wt % to *n*BMA, whereas at higher SDS amounts, no coagulation occurred. A series of reactions were successfully performed using 4.6 wt % SDS to *n*BMA (Figure 1). Good control over the process was noticed even with a low catalyst loading (100 ppm). An excess of TPMA as an electron donor was used to photochemically (re)generate the Cu activator. Additionally, an auxiliary electrolyte as an ion carrier was applied [34].

The external regulation of the polymerization reaction was presented by switching the UV light on and off resulting in temporal control over the process. The versatility of this system was demonstrated by successful polymerization of *n*BMA and *n*BA from small-molecular initiators as well as a macroinitiator [34].

In comparison to the initial work in the field of photo-ATRP, Matyjaszewski et al. went a step further, using the system in ab initio emulsion with the generation of radicals in the aqueous phase, followed by nucleation of particles and stabilization by surfactant molecules [35]. The growing particles are continuously replenished by monomer molecules that diffuse through the aqueous phase from big monomer droplets. Therefore, after the initial nucleation stage, the polymerization proceeds inside the hydrophobic polymer particles [35]. The optimized photoinduced ATRP polymerization system in the emulsion with a low surfactant concentration and no co-surfactant is the preferable environment for industrial polymerizations.

## 5. Ultrasound-Mediated Atom Transfer Radical Polymerization (sono-ATRP)

**Scheme 7:**
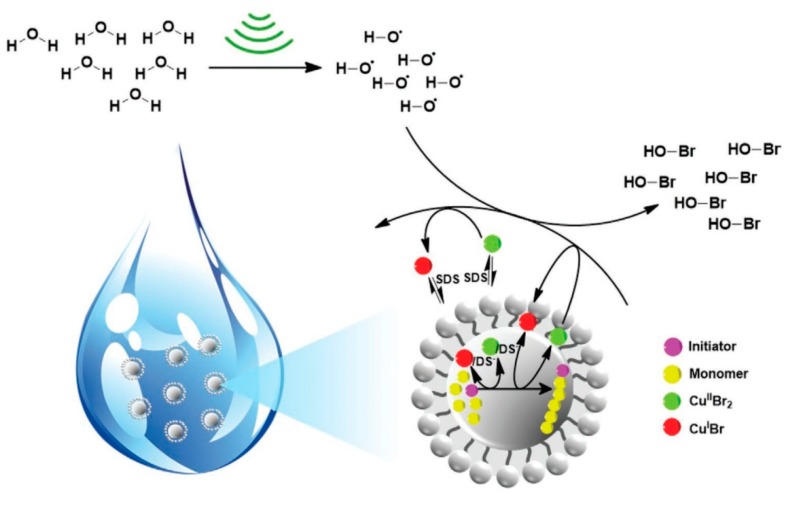
The concept of ion-pair and interfacial catalysis in ultrasound-mediated ATRP conducted in miniemulsion media. Reprinted with permission from reference [43], WILEY-VCH Verlag GmbH and Co. KGaA, Weinheim. Copyright 2019.

Particular attention in the context of ATRP in dispersion systems is attracted by the recently published ultrasonication-mediated atom transfer radical polymerization (sono-ATRP) in a miniemulsion environment with a reduced concentration of a catalyst complex [43]. The ultrasonic-induced technique in dispersed media eliminates using an additional reducing agent. This technique was applied for the first time with the use of a strongly hydrophilic catalyst complex (Cu^II^Br_2_/TPMA) in the reaction setup. This composition was responsible for two crucial mechanisms—interfacial and ion-pair catalysis in accordance with single-catalyst mechanism (Scheme 7) [38,43].

Sono-ATRP provides polymerization of well-defined polymers with preservation of chain-end functionality and a narrow molecular weight distribution by using an external stimulus. Moreover, temporal control in miniemulsion sono-ATRP is also possible by turning on/off the ultrasonication without having an adverse influence on the precisely-defined, receiving polymers with low dispersity and preserved chain-end fidelity. Considering the “green chemistry” aspect, as well as the results of experiments and the simplicity of scaling reaction setup, this procedure constitutes a simple and eco-friendly approach to obtain functional polymeric materials [43]. Table 4 shows the synthesis results obtained by the sono-ATRP approach in minemulsion using different monomers and reaction initiators.

The application of anionic surfactant (SDS) with the presence of catalyst copper(II) bromide/tris(2-pyridylmethyl) amine provided a single catalyst system. The series of syntheses of P*n*BA and poly(methyl methacrylates) (PMMA) homopolymers and copolymers was successfully conducted, and products with narrow molecular weight distribution were obtained. The polymerization of PMMA initiated by ethyl *α*-bromophenylacetate (EBPA) gave polymers with wider distribution (*Đ* = 1.6) Although 100% conversion has not been achieved in any reaction, this method is promising for industrial applications due to its easy scalability and reduced amount of reagents. However, it requires further optimization to increase conversion and reduce the response time while maintaining well-defined final products. The ultrasonication-mediated system based on the ion-pair and interfacial catalysis without the application of additional reducing agent can be considered an interesting and promising possibility in the field of ATRP [43].

## 6. Conclusion and Future Prospective

Over the past decade there has been significant progress in ATRP techniques. The lower catalytic complex quantities in aqueous dispersed media wereaccomplished, especially beneficial in microelectronic and biomedical applications. These methods in water dispersed media were conducted using (ultra)low ppm catalyst loading: *e*ATRP (321–1,000 ppm), ARGET ATRP (50–2,000 ppm), photo-ATRP (100–800 ppm) and sono-ATRP (585–717 ppm). All of the mentioned ATRP techniques enable the use of a low quantity of catalyst accompanied by a good control over the polymerization and the preparation of well-defined final products with a narrow molecular weight distribution. In this context, the proper development path will be the complete elimination of the catalytic complex from the reaction system. This type of concept was implemented in the metal-free approach according to the Pickering emulsion [46]; however, it is still a challenge in relation to miniemulsion approach with the elimination of additional reagents such as photoinitiator. It is worth emphasizing that using water as a polymerization media is useful only when products can be obtained which do not fall into the environmental contaminating classification. The (mini)emulsion ATRP with highly toxic components, such as the copper salts and ligands, has not been yet utilized by industry at all yet; therefore, the development of ATRP polymerization towards being metal-free and the replacement of toxic substances by bio-based multi-tasking compounds is very important. Appropriate development of polymerization in biphasic systems should focus on limiting the use of surfactants and co-surfactants, replacing them with multifunctional molecules, acting simultaneously as a monomer or an initiator of polymerization for either ligand of the catalytic complex. Polymerizations without additional reagents, such as reducing agent, initiator and catalytic complex, can be possible by using multi-tasking structures performing several functions at the same time. The excellent example of such a solution is riboflavin-based macromolecule, which has structural elements excited by energy quantum, allowing for successful metal-free polymerization [56]. The polymer materials obtained in this way can be used without hesitation as elements of microelectronics and in the field of biomedical and biotechnological applications, in particular for the needs of tissue engineering and for the production of medical implants.

## Figures and Tables

**Table 1:** *e*ATRP of *n*BA in miniemulsion with different surfactants and catalysts ^a^.

Entry	Monomer (% vol)	Initiator	(Cu^II^Br_2_)/L_aq_/L_org_		Surfactant (% wt to monomer)	*E*_app_	Catalyst Concentration		*Đ*	Ref.
			L_aq_	L_org_			Ppm [CuL]/[M]	Ppm (by wt)		
**1**	*n*BA (20)	EBiB	BPY	-	SDS (4.6)	*E*_pc_	1000	2,503	9.30	[76]
2	*n*BA (20)	EBiB	BPMEA	-	SDS (4.6)	*E*_pc_	1000	2,503	4.62	[76]
3	*n*BA (20)	EBiB	BPY	BPMODA	SDS (4.6)	*E*_pc_	1000	2,503	1.78	[76]
4	*n*BA (20)	EBiB	BPMEA	BPMODA	SDS (4.6)	*E*_pc_	1000	2,503	1.50	[76]
5	*n*BA (20)	EBiB	TPMA	BPMODA	SDS (4.6)	*E*_pc_	1000	2,503	2.53	[76]
6	*n*BA (20)	EBiB	BPY	BPMODA*	SDS (4.6)	*E*_pc_	1000	2,503	1.26	[76]
7	*n*BA (20)	EBiB	BPMEA	BPMODA*	SDS (4.6)	*E*_pc_	1000	2,503	1.19	[76]
8	*n*BA (20)	EBiB	TPMA	BPMODA*	SDS (4.6)	*E*_pc_	1000	2,503	1.32	[76]
9	*n*BA (20)	EBiB	TPMA	-	Brij-98 (6.2)	*E*_pc_-0.03V	-	2,181	4.77	[38]
10	*n*BA (20)	EBiB	TPMA	-	SDS (6.2)	*E*_pc_-0.03V	321–720	2,181	1.09–1.26	[38]
11	*n*BA (20)	EBiB	TPMA *^2^	-	SDS (6.2)	*E*_pc_-0.03V	-	2,181	1.32	[38]
12	*n*BA (20)	EBiB	Me_6_TREN	-	SDS (6.2)	*E*_pc_-0.03V	-	2,181	1.94	[38]

^a^*n*-hexadecane (HD) was used as cosurfactant and NaBr as supporting electrolyte.

**Table 2:** ARGET ATRP in miniemulsion with different surfactants and catalysts ^a^.

Entry	Monomer (% vol)	Initiator	(Cu^II^Br_2_)/L_aq_/L_org_		Surfactant (% wt to Monomer)	Reducing Agent	Temp.	Catalyst Concentration		*Đ*	Ref.
			L_aq_	L_org_				Ppm [CuL]/[M]	Ppm (by wt)		
1	*n*BA (20)	EBiB	-	BPMODA*	Brij-98 (2.3)	Sn(EH)_2_	80 °C	250–2,000	670 ^b^	1.15–1.51	[86]
2	*n*BMA (20)	EBPA	-	DOD-BPED*	Brij-98 (2.3)	AsAc	80 °C	50–250	-	1.23–1.39	[87]
3	*n*BA (20)	EBiB	TPMA	-	SDS (1.15–9.2)	AsAc	65 °C	144–719	216 ^c^	-	[19]
4	*n*BMA (20)	EBPA	TPMA	-	SDS (4.6)	AsAc	65 °C	50–800	216 ^c^	1.2–1.42	[19]
5	*n*BMA (20)	EBPA	BPMODA*	-	SDS (4.6)	AsAc	65 °C	800	-	1.18	[19]
6	*n*BA (20)	EBiB/EBPA	TPMA	-	SDBS (4.6)	AsAc	65 °C	704	216 ^c^	-	[19]
7	*n*BA (20)	EBiB/EBPA	TPMA	-	SDS+SDA (4.6 + 0.5)	AsAc	65 °C	704	216 ^c^	-	[19]
8	*n*BA (17)	Rib-Br_2_	TPMA	-	SDS (6.2)	Rib-Br_2_	65 °C	700–1,000	186–267	1.39–2.05	[66]
9 ^d^	*n*BA (17)	Rib-Br_2_	TPMA	-	SDS (6.2)	Rib-Br_2_	65 °C	1,000	266	1.19	[66]

^a^*n*-hexadecane (HD) was used as cosurfactant and NaBr as supporting electrolyte ^b^ for reaction with 2000 ppm of Cu^II^Br_2_, ^c^ according to Table 1 at reference [19]; ^d^ reaction in air conditions.

**Table 3:** Photo-ATRP in miniemulsion with different catalysts and monomers ^a^.

Entry	Monomer (% vol)	Initiator	Cu^II^Br_2_/L_aq_	Surfactant (% wt to monomer)	λ (nm), Intensity (mW/cm^2^)	Catalyst Concentration		*Đ*	Ref.
						Ppm [CuL]/[M]	Ppm (by wt)		
1	*n*BMA (5-50)	EBPA	TPMA	SDS (1.2–18.4)	370 (5)	800–100	220	1.24–1.76	[34]
2	*n*BA (20)	EBiB	TPMA	SDS (4.6)	370 (5)	800	220	1.3	[34]
3	MMA (20)	PEO_2K-_BPA	TPMA	SDS (18.4)	394 (2.6)	400	-	1.75	[35]
4	EMA (20)	PEO_2K-_BPA	TPMA	SDS (18.4)	394 (2.6)	400	-	1.24	[35]
5	*n*BMA (20)	PEO_2K-_BPA	TPMA	SDS (18.4)	394 (2.6)	400	190	1.09	[35]
6	LMA (20)	PEO_2K-_BPA	TPMA	SDS (18.4)	394 (2.6)	400	-	-	[35]
7	*n*BA (20)	PEO_2K-_BiB	TPMA	SDS (18.4)	394 (2.6)	400	-	1.13	[35]

^a^*n*-hexadecane (HD) was used as cosurfactant in entries 1 and 2, NaBr as supporting electrolyte except entries 3-7: NaBr/TEA as supporting electrolyte.

**Table 4:** Sono-ATRP in miniemulsion with different catalysts and monomers ^a^.

Entry	Monomer (% vol)	Initiator	Cu^II^Br_2_/L_aq_	Surfactant (% wt tom)	Frequency of Sonication (kHz)	Catalyst Concentration		*Đ*	Ref.
						Ppm [CuL]/[M]	Ppm (by wt)		
1	*n*BA (20)	EBiB	TPMA	SDS (6.2)	40	717	189	1.26–1.28	[43]
2	MMA (20)	EBiB	TPMA	SDS (6.2)	40	717	244	1.27	[43]
3	MMA (20)	EBPA	TPMA	SDS (6.2)	40	717	243	1.6	[43]
4	*n*BA (20)	P*n*BA-Br	TPMA	SDS (6.2)	40	585	621	1.41	[43]
5	*t*BA (20)	P*n*BA-Br	TPMA	SDS (6.2)	40	588	623	1.27	[43]

^a^*n*-hexadecane (HD) was used as cosurfactant and NaBr as supporting electrolyte, reactions temp. 65 °C.

## Abbreviations (alphabetical order)

ARGET ATRP	activators regenerated by electron transfer atom transfer radical polymerization
AsAc	ascorbic acid
ATRP	atom transfer radical polymerization
*n*BA	*n*-butyl acrylate
*n*BMA	*n*-butyl methacrylate
BPMEA	*N,N*-bis(2-pyridylmethyl)-2-hydroxyethylamine
BPMODA	bis(2-pyridylmethyl)octadecylamine
BPMODA*	bis[2-(4-methoxy-3,5-dimethyl)pyridylmethyl]octadecylamine
BPY	2,2′-bipyridine
Brij-98	polyoxyethylene(20) oleyl ether
DOD-BPED*	*N,**N*-dioctadecyl-*N,**N*-bis[2-(4-methoxy-3,5-dimethyl)pyridylmethyl]ethane-1,2-diamine
*e*ATRP	electrochemically mediated atom transfer radical polymerization
EBiB	ethyl α-bromoisobutyrate
EBPA	ethyl α-bromophenylacetate
EMA	ethyl methacrylate
*E*_pc_	cathodic peak potential
ICAR ATRP	initiators for continuous activator regeneration atom transfer radical polymerization
LMA	lauryl methacrylate
Me_6_TREN	tris(2-(dimethylamino)ethyl)-amine
mechano-ATRP	mechanically induced atom transfer radical polymerization
MMA	methyl methacrylate
MWD	molecular weight distribution
P*n*BA	poly(*n*-butyl acrylate)
P*n*BMA	poly(*n*-butyl methacrylate)
P*t*BMA	poly(*t*-butyl methacrylate)
PEO_2K-_BiB	poly(ethlene glycol) 2-bromoisobutyrate
PEO_2K-_BPA	poly(ethylene glycol) 2-bromophenylacetate
photo-ATRP	photoinduced atom transfer radical polymerization
PMMA	poly(methyl methacrylate)
Rib-Br_2_	brominated riboflavin
SARA ATRP	supplemental activator and reducing agent atom transfer radical polymerization
SDA	sodium dodecanoate
SDBS	sodium dodecylbenzenesulfonate
SDS	sodium dodecyl sulfate
Sn(EH)_2_	tin(II) 2-ethylhexanoate
sono-ATRP	ultrasound-mediated atom transfer radical polymerization
*t*BA	*tert*-butyl acrylate
TEA	triethylamine
TPMA	tris(2-pyridylmethyl)amine
TPMA*^2^	1-(4methoxy-3,5-dimethylpyridin-2-yl)-*N*-((4-methoxy-3,5-dimethylpyridin-2-yl)methyl)-*N*-(pyridin-2-ylmethyl)methanamine
